# Renal medullary (pro)renin receptor contributes to angiotensin II-induced hypertension in rats via activation of the local renin–angiotensin system

**DOI:** 10.1186/s12916-015-0514-1

**Published:** 2015-11-10

**Authors:** Fei Wang, Xiaohan Lu, Mi Liu, Yumei Feng, Shu-Feng Zhou, Tianxin Yang

**Affiliations:** Institute of Hypertension, Sun Yat-sen University School of Medicine, #74 Zhongshan 2nd Road, Science and Technology Building, 6th Floor, Guangzhou, 510080 P. R. China; Internal Medicine, University of Utah and Veterans Affairs Medical Center, Salt Lake City, UT USA; Departments of Pharmacology and Physiology & Cell Biology, University of Nevada School of Medicine, Reno, NE USA; Department of Pharmaceutical Sciences, College of Pharmacy, University of South Florida, Tampa, FL USA

**Keywords:** Angiotensin II, PRO20, (Pro)renin receptor, Renin activity, Vascular smooth muscle cells

## Abstract

**Background:**

(Pro)renin receptor (PRR) is a new component of the renin–angiotensin system and regulates renin activity in vitro. Within the kidney, PRR is highly expressed in the renal medulla where its expression is induced by angiotensin II infusion. The objective of the present study was to test a potential role of renal medullary PRR during angiotensin II-induced hypertension.

**Methods:**

A rat AngII infusion model (100 ng/kg/min) combined with renal intramedullary infusion of PRO20, a specific inhibitor of PRR, was builded. And the intravenous PRO20 infusion serve as control. Mean arterial pressure was recorded by radiotelemetry for one week. Further anaylsis of kidney injury, inflammation, biochemical indices and protein localization were perrformed in *vivo* or in *vitro*.

**Results:**

Radiotelemetry demonstrated that AngII infusion elevated the mean arteria pressure from 108 ± 5.8 to 164.7 ± 6.2 mmHg. Mean arterial pressure decreased to 128.6 ± 5.8 mmHg (*P* < 0.05) after intramedullary infusion of PRO20, but was only modestly affected by intravenous PRO20 infusion. Indices of kidney injury, including proteinuria, glomerulosclerosis, and interstitial fibrosis, inflammation, and increased renal medullary and urinary renin activity following angiotensin II infusion were all remarkably attenuated by intramedullary PRO20 infusion. Following one week of angiotensin II infusion, increased PRR immunoreactivity was found in vascular smooth muscle cells. In cultured rat vascular smooth muscle cells, angiotensin II induced parallel increases in soluble PRR and renin activity, and the latter was significantly reduced by PRO20.

**Conclusion:**

Renal medullary PRR mediates angiotensin II-induced hypertension, likely by amplifying the local renin response.

## Background

The renin–angiotensin system (RAS) is one of the most important regulatory systems for the control of extracellular volume and blood pressure (BP). Over-activation of the RAS plays an essential role in the pathogenesis of hypertension as evidenced by the wide use of angiotensin-converting enzyme (ACE) inhibitors and AT1-receptor antagonists for the management of human hypertension [1–3]. In humans and animals, activation of the RAS due to renal artery stenosis leads to profound hypertension and cardiovascular morbidity [4]. Angiotensin II (AngII), the major effector hormone of the RAS, when given at a pressor dose, readily induces hypertension [5]. However, despite intensive investigation, the mechanism of AngII-induced hypertension is still incompletely understood. Although diverse mechanisms have been proposed, increasing evidence suggests involvement of the local RAS found in a variety of tissues, including the brain, heart, adrenal gland, vasculature, and kidney [6, 7]. Over the past decade, there has been a paradigm change in our understanding of the potential role of the local versus systemic RAS in the pathogenesis of hypertension. In particular, mounting evidence is available to support an essential role of the intrarenal RAS in AngII-induced hypertension.

(Pro)renin receptor (PRR) was cloned as a specific receptor for prorenin and renin by Nguyen et al. in 2002 [8]. It is a 350-amino-acid protein containing a single transmembrane domain [9]. A soluble form of PRR (sPRR), that is, the N-terminal domain fragment, is generated by intracellular cleavage by furin and secreted in plasma [10]. The carboxyterminal tail has previously been purified from chromaffin granules as an 8–9-kDa accessory protein (M8-9) of the vacuolar-type H^+^-ATPase and designated ATP6AP2 [11]. In vitro evidence demonstrates that prorenin bound to PRR has increased catalytic activity, thus mediating local AngII formation [8, 12]. In light of its ubiquitous expression in a variety of tissues, PRR is postulated to function as a regulator of tissue renin activity [13]. However, there is no convincing in vivo evidence to prove the renin-regulatory function of PRR. Apart from prorenin or renin activation, activation of PRR by (pro)renin stimulates a variety of signal transduction pathways such as mitogen-activated protein kinase [14] and Wnt-β-catenin pathways [15], independent of AngII [14, 15]. Within the kidney, high levels of PRR immunoreactivity have been detected in intercalated cells of the collecting duct (CD) [16, 17] as well as vascular smooth muscle cells (VSMC) [8]. Previous studies from us and others have shown that PRR expression in the kidney, particularly in the renal medulla, increases during AngII-induced hypertension, dependent of the COX-2/EP4 pathway [16, 18–20]. The present study tested the functional role of renal medullary PRR during AngII-induced hypertension.

## Methods

### Rat experiments

Male Sprague-Dawley rats (220–250 g, Charles River Laboratories, Wilmington, MA, USA) were cage-housed and maintained in a temperature-controlled room with a 12:12-h light–dark cycle, with free access to tap water and standard rat chow for 14 days. The animal protocols were approved by the Animal Care and Use Committees at Sun Yat-sen University and University of Utah. The rats underwent uninephrectomy with or without bilateral adrenalectomy and were instrumented with radiotelemetric devices. After one week’s recovery from the surgery, a second surgery was performed to place a subcutaneous osmotic mini-pump delivering vehicle or AngII at 100 ng/kg/min. In this surgery, the kidney was exposed from the flank region and a catheter was placed in the renal medulla, approximately 4.0 mm underneath the surface, and secured using Vetbond glue; the other end of the catheter was connected to an osmotic mini-pump delivering vehicle or PRO20 at 120 μg/kg/d. A separate group of rats received intravenous infusion of PRO20 via the jugular vein and served as a control. Adrenalectomized rats were given dexamethasone at 1 mg/ml in their drinking water starting 2 days prior to adrenalectomy until the end of the experiment. The radiotelemetric device was implanted via catheterization of the carotid artery and was turned on for 4 h per day from 5:00 p.m. to 9:00 p.m. The data from the rats with incorrectly positioned intramedullary infusion catheters detected at sacrifice were excluded from the final analysis.

### Biochemical analysis of renin

Renin activity in plasma, urine, tissue homogenates, and cell culture medium was determined under the native condition by measurement of AngI generation using enzyme-linked immunosorbent assay (ELISA). Aldosterone concentrations in plasma, urine, and cell culture medium were measured using a commercial ELISA kit (Cat#:10004377, Cayman Chemical, Ann Arbor, Michigan, USA).

### Enzyme immunoassay

To detect urinary or medium prorenin/renin, sPRR, we used the following commercially available enzyme immunoassay kits according to the manufacturer’s instructions: prorenin/renin (Molecular Innovations, Novi, MI, USA) and sPRR (IBL, Toronto, Canada).

### Renal histology

Under anesthesia, kidneys were harvested and fixed with 10 % paraformaldehyde. The tissues were subsequently embedded in paraffin and 4-μm sections were cut and stained with periodic acid–Schiff. Renal pathologies including glomerulosclerosis and interstitial fibrosis were scored on a 1–4 scale as previously described [21] (the higher the number, the more severe the injury).

### Quantitative reverse transcriptase polymerase chain reaction

For Quantitative reverse transcription polymerase chain reaction (qRT-PCR), total RNA isolation and reverse transcription were performed as previously described [22]. Oligonucleotides were designed using Primer3 software (available at http://bioinfo.ut.ee/primer3-0.4.0/). Primers for TNF-α were 5′- CCACGTCGTAGCAAACCACCAAG-3′ (sense) and 5′- CAGGTACATGGGCTCATACC-3′ (antisense); primers for IL-18 were 5′-TGGAGACTTGGAATCAGACC-3′ (sense) and 5′-GGCAAGCTAGAAAGTGTCCT-3′ (antisense); primers for GAPDH were 5′-GTCTTCACTACCATGGAGAAGG-3′ (sense) and 5′-TCATGGATGACCTTGGCCAG-3′ (antisense).

### Immunofluorescence staining

The tissues were fixed in 10 % neutral buffered formalin for 24 h and then embedded in paraffin. After deparaffinization, thin sections (4 μm) were processed for double-labeling with immunofluorescence. The slides were blocked in 1 % bovine serum albumin for 1 h and were then incubated with primary antibody at 4 °C overnight. After washing off the primary antibody, sections were incubated for 1 h at room temperature with Donkey anti-goat-IgG- fluorescein isothiocyanate (1:75, sc-2024, Santa Cruz Biotechnology, Santa Cruz, CA, USA) or donkey anti-rabbit IgG-tetramethylrhodamine (1:100, A31572, Life Technologies, Grand Island, NY, USA). Rabbit anti-PRR antibody was raised against residues 335–350 in the C terminus (Cat#: ab40790, Abcam, Cambridge, MA, USA). Mouse anti-α-smooth muscle actin (α-SMA) antibody was purchased from Sigma (Cat#: F3777, Sigma, St Louis, MO, USA).

### Cell culture

Rat aorta smooth muscle cell line (VSMC) was purchased from ATCC, Manassas, VA, USA (Cat# CRL-2018) and grown in a six-well plate. After the cell monolayers reached 95 % confluence, the VSMCs were pretreated with PRO20 (1.5 μM) for 1 h, followed by AngII treatment at 100 nM for 24 h. After the treatment, the medium was collected for enzyme assays or renin assays.

### Statistical analysis

Data is summarized as means ± standard error (SE). All data points were included in the statistical analyses. Sample sizes were determined on the basis of similar previous studies or pilot experiments. Statistical analysis for animal and cell cultures experiments was performed using analysis of variance with the Bonferroni test for multiple comparisons or by paired or unpaired Student’s *t*-test for two comparisons. A *P*-value below 0.05 was considered statistically significant.

## Results

### Pharmacological investigation of renal medullary function of PRR

**Fig. 1:**
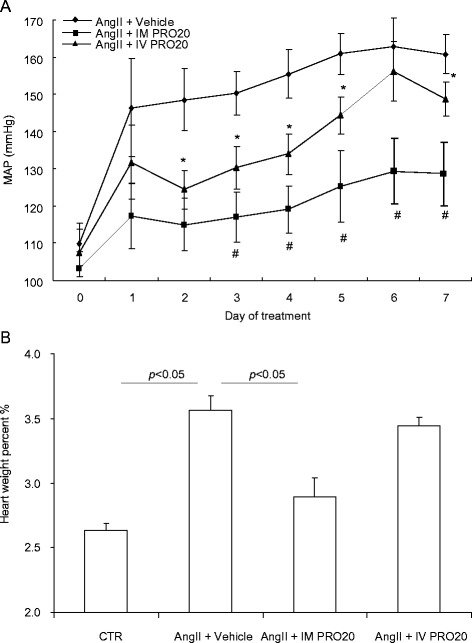
Effect of intramedullary (pro)renin receptor (*PRR*) inhibition on angiotensin II (*AngII*)-induced hypertension in rats. Uninephrectomized male Sprague-Dawley rats were divided into the following three groups: (1) AngII, (2) AngII + intramedullary PRO20 infusion (*IM PRO20*), and (3) AngII + intravenous PRO20 infusion (*IV PRO20*). AngII was subcutaneously infused at 100 ng/kg/min via an osmotic mini-pump. IM PRO20 (PRO20 at 120 μg/kg/d) was performed via a catheter chronically implanted in the renal medulla. To control the spillover, IV PRO (PRO20 at 120 μg/kg/d) was performed via catheterization of the jugular vein. Telemetry was performed to monitor mean arterial pressure (*MAP*) and it was turned on 4 h per day from 5:00 p.m. to 9:00 p.m. for 7 days. **a** Radiotelemetry monitoring of MAP. ^#^ *P* < 0.01 versus intravenous PRO20; *P < 0.05 versus AngII alone. **b** Cardiac hypertrophy. Heart weight is expressed as percentage of body weight. Control (*CTR*), N = 6; AngII + Vehicle, N = 9; AngII + IM PRO20, N = 8; AngII + IV PRO20, N = 6. Data are mean ± standard error

PRO20 is a newly developed 21-amino-acid PRR decoy peptide that interrupts the binding of prorenin to PRR with high potency and specificity [23]. To probe the functional role of renal medullary PRR, we employed an intramedullary infusion technique that allows site-specific delivery of an agent to the renal medulla [24]. To this end, a catheter was chronically implanted in the renal medulla of nephrectomized rats to achieve site-specific delivery of PRO20, and intravenous infusion of this peptide via the jugular vein served as a control for spillover. Radiotelemetry was used to monitor daily mean arterial pressure (MAP). One-week AngII infusion induced immediate and sustained increases in MAP, from 108 ± 5.8 (day 0) to 164.7 ± 6.2 mmHg (day 7) (Fig. 1a). Intramedullary PRO20 infusion (IM PRO20) remarkably attenuated AngII-induced hypertension and lowered the MAP to 128.6 ± 5.8 mmHg. However, intravenous PRO20 infusion (IV PRO20) was much less effective than IM PRO20 in lowering MAP (Fig. 1a). Consistent with the BP data, AngII-induced cardiac hypertrophy was blunted by IM PRO20 but not IV PRO20 (Fig. 1b).

**Fig. 2:**
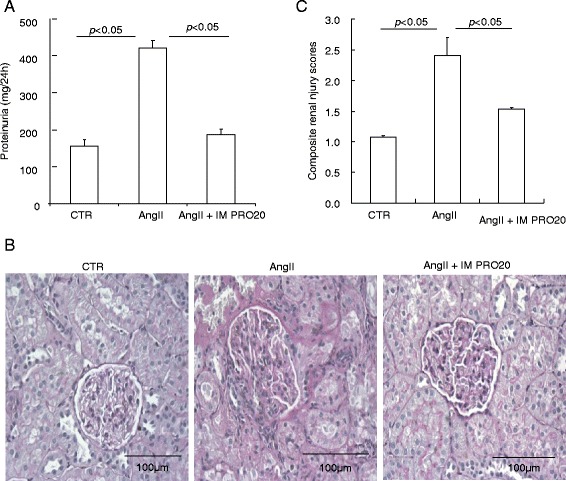
Effect of intramedullary (pro)renin receptor (*PRR*) inhibition on angiotensin II (*AngII*)-induced kidney injury in rats. **a** Measurement of urinary protein excretion using Coomassie blue. **b** Representative micrographs of periodic acid–Schiff staining of kidney sections. **c** Renal injury scores from semi-quantitative analysis of renal pathologies. N = 6–14 per group. Data are mean ± standard error. *CTR* control, *IM PRO20* intramedullary PRO20 infusion, *IV PRO20* intravenous PRO20 infusion

**Fig. 3:**
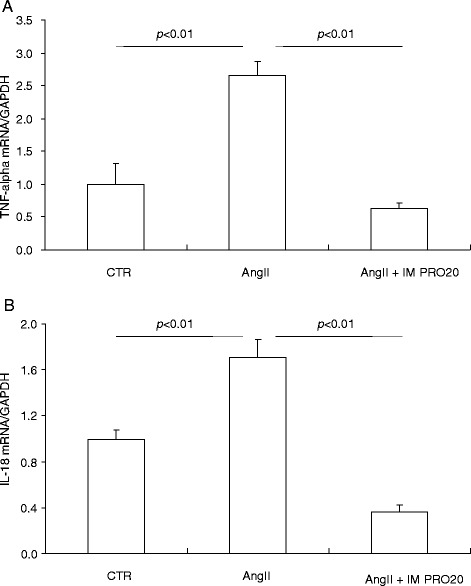
Effect of intramedullary (pro)renin receptor (*PRR*) inhibition on renal inner medullary expression of cytokines during angiotensin II (*AngII*)-induced hypertension in rats. **a**, **b** The expression of tumor necrosis factor alpha (*TNF-α*) and interleukin 18 (*IL-18*) in the inner medulla of control (*CTR*), AngII, and AngII + intramedullary PRO20-infused (*AngII + IM RPO20*) rats was determined by quantitative reverse transcriptase polymerase chain reaction, which is normalized by glyceraldehyde 3-phosphate dehydrogenase (*GAPDH*). N = 6 per group. Data are mean ± standard error

Following AngII infusion, the uninephrectomized rats developed severe kidney injury as evidenced by increased proteinuria (Fig. 2a) and renal histological changes, including glomerulosclerosis and interstitial fibrosis (Fig. 2b, c). These indices of kidney injury were all attenuated by IM PRO20 (Fig. 2a–c). An inflammatory response is a well known important feature of AngII-induced hypertension [25, 26]. Therefore, we examined renal expression of inflammatory markers such as TNF-α and IL-18 using qRT-PCR. Both cytokines were elevated by AngII infusion and were blunted by IM PRO (Fig. 3a, b). PRO20 treatment via intramedullary or intravenous infusion was not associated with any noticeable toxicity.

**Fig. 4:**
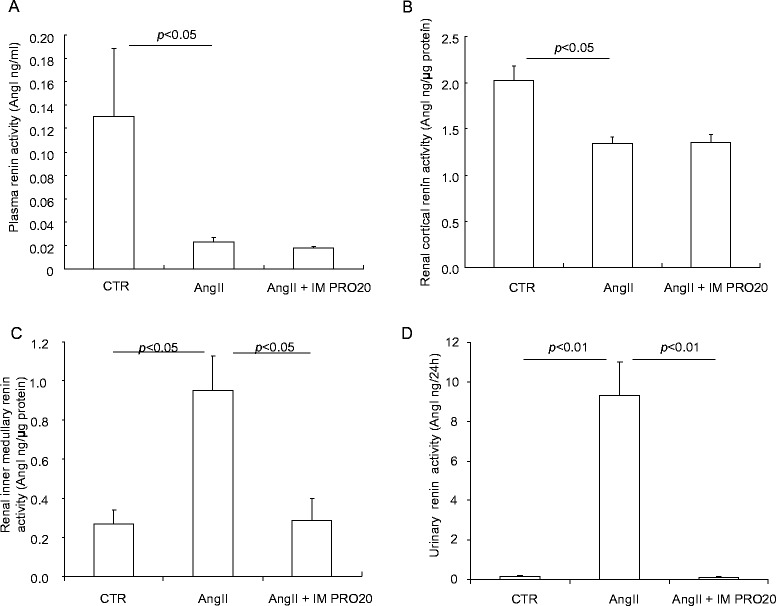
Effect of intramedullary (pro)renin receptor (*PRR*) inhibition on renin levels in angiotensin II (*AngII*)-infused rats. Plasma, urine, and renal tissues from control (*CTR*), AngII, and AngII + intramedullary PRO20-infused (*AngII + IM RPO20*) rats were assayed for renin activity by measurement of AngI generation. **a** Plasma renin activity. **b** Renal cortical renin activity. **c** Renal inner medullary renin activity. **d** Urinary renin activity. N = 5–6 per group. Data are means ± standard error

**Fig. 5:**
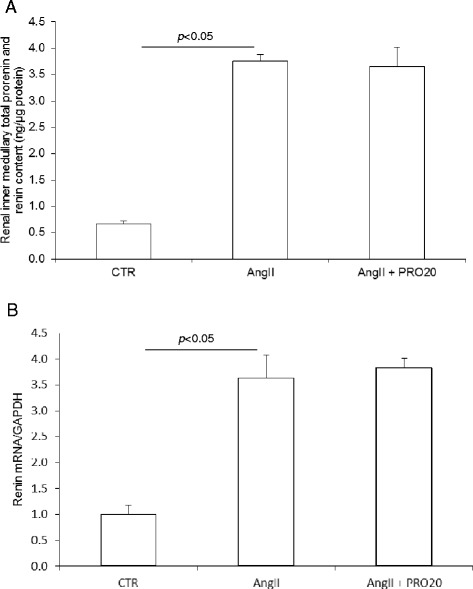
Effect of intramedullary (pro)renin receptor (*PRR*) inhibition on renal inner medullary prorenin/renin expression in angiotensin II (*AngII*)-infused rats. The expression of prorenin/renin in the inner medulla was determined by using enzyme-linked immunosorbent assay (ELISA) and quantitative reverse transcriptase polymerase chain reaction (qRT-PCR) determined in Control (*CTR*), AngII, or AngII + intramedullary PRO20-infused (*AngII + IM RPO20*) rats. **a** ELISA detection of prorenin/renin content. **b** qRT-PCR detection of renin mRNA expression. N = 5–6 per group. Data are means ± standard error

AngII infusion induces a local renin response in the renal medulla but suppresses systemic renin activity [16, 18, 27–29]. We hypothesized that PRR may be required to enhance the renal medullary renin response to AngII. As expected, following AngII infusion, renin activity was suppressed in plasma and the renal cortex but enhanced in the inner medulla and urine, and the latter response was blunted by IM PRO20 (Fig. 4a–d). ELISA showed that prorenin/renin content in the inner medulla was elevated by AngII infusion but was unaffected by IM PRO20 (Fig. 5a) and the same result was obtained by qRT-PCR of renin mRNA (Fig.5b), supporting the concept that PRR primarily regulates local renin activity but not renin expression. Of note, the ELISA kit was unable to differentiate between prorenin and renin.

### Regulation and function of vascular PRR

**Fig. 6:**
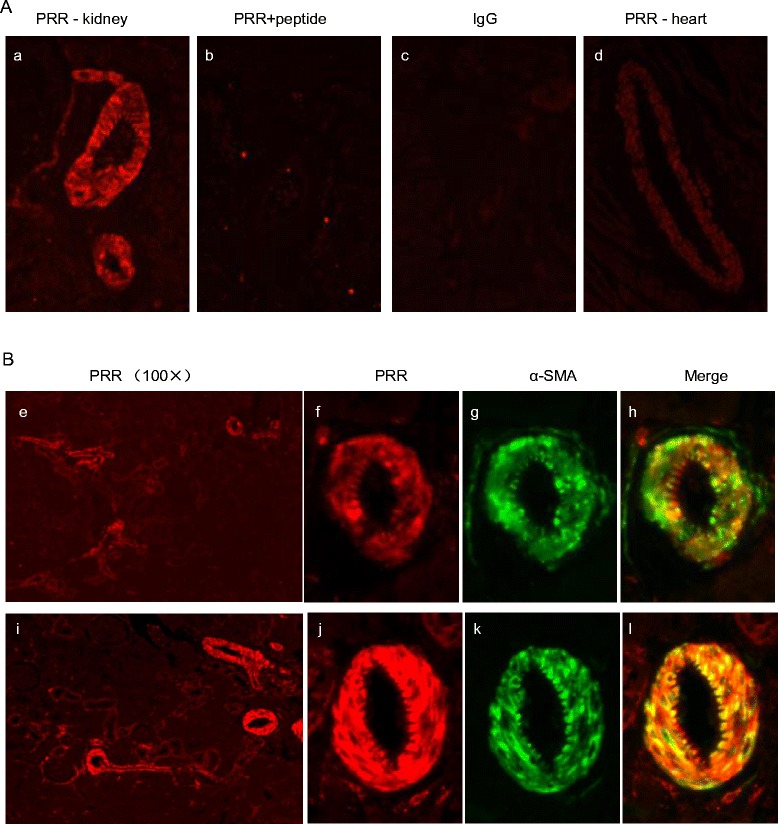
Immunostaining analysis of renal (pro)renin receptor (*PRR*) expression during angiotensin II (AngII)-induced hypertension. **a** Validation of specificity of the labeling. (*a*) Immunostaining of PRR antibody in kidney; (*b*) Immunostaining in the kidney in the presence of the immune PRR peptide (Cat#ab41522, Abcam); (*c*) Immunostaining of the kidney with rabbit IgG protein only; (*d*) Immunostaining of PRR in the heart. **b** Co-labeling with anti-PRR antibody and anti-α-SMA antibody in control group (*e*–*h*) and AngII group (*i*–*l*). The kidney section was stained with anti-PRR antibody and anti-α-SMA antibody (*e*–*g*; *i*–*k*). The merged images are shown in (*h*) and (*l*). (*e*) and (*i*): 100× magnification; (*f*–*h*) and (*j*–*i*): 400× magnification. Shown are representatives of three to six animals per group

**Fig. 7:**
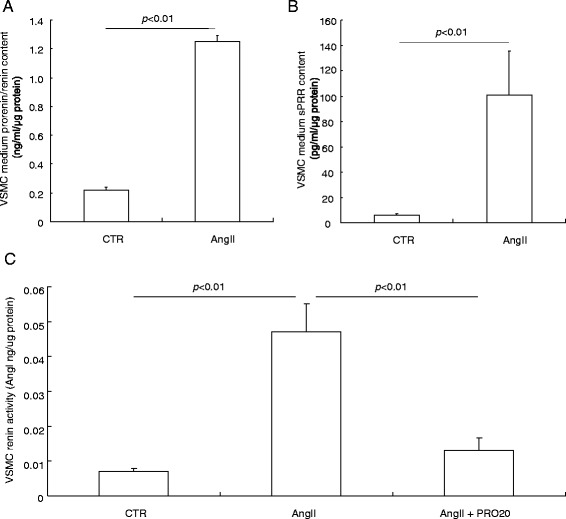
Effect of angiotensin II (*AngII*) on prorenin/renin content, soluble (pro)renin receptor (*sPRR*) content and renin activity in rat vascular smooth muscle cells (*VSMCs*). The cells were exposed to 100 nM AngII for 24 h and medium prorenin/renin content (**a**) and sPRR content (**b**) was analyzed by enzyme-linked immunosorbent assay. Medium renin activity (**c**) was analyzed by measurement of AngI generation in the absence of substrate. N = 12 per group. Data are means ± standard error. *CTR* control

Immunostaining revealed the strongest signal of PRR in the vascular smooth muscle in the kidney (Fig. 6a). This signal was specific because it was completely eliminated by the immunizing peptide (Fig. 6a). The vascular labeling of PRR appeared weaker in the heart as compared to that in the kidney (Fig. 6a). AngII infusion enhanced the vascular labeling of PRR in the kidney (Fig. 6b). PRR labeling was co-localized with α-SMA labeling (Fig. 6b). In cultured rat VSMC, exposure to 100 nM AngII for 24 h induced a marked increase in medium prorenin/renin and sPRR, both being assessed by ELISA (Fig. 7a). In parallel, renin activity was increased, as reflected by measuring AngI generation, and was blunted by PRO20 (Fig. 7b).

## Discussion

The importance of the RAS in the pathogenesis of human hypertension is highlighted by the wide use of ACE inhibitors and angiotensin receptor blockers as first-line antihypertensive therapies [1–3]. However, despite intensive investigation, the mechanism of AngII-induced hypertension is still incompletely understood. We used a pharmacological approach to investigate the functional role of renal medullary PRR during AngII-induced hypertension. PRO20 is a newly developed, highly specific PRR decoy inhibitor that interrupts the binding of prorenin to PRR [30]. The inhibitor was directly delivered to the rat renal medulla to evaluate the contribution of renal medullary PRR to AngII-induced hypertension. The result showed that renal delivery of PRO20 almost completely abolished AngII-induced hypertension, contrasting with a relatively modest BP-lowering effect of IV PRO20. The difference in the effects of local versus systemic delivery of PRO20 reflects the contribution of renal medullary PRR.

Although abundant in vitro evidence demonstrates that PRR binds renin and prorenin to increase their catalytic activity [8, 12, 31–33], solid in vivo evidence to support PRR as a renin regulator is still lacking. In fact, increasing skepticism has surrounded the renin-regulatory function of PRR. For example, overexpression of human PRR in rats resulted in proteinuria and nephropathy but did not elevate BP or renal AngII levels [34, 35]. The lack of viable PRR null mice and an effective PRR inhibitor has made it difficult to convincingly prove PRR as a key player in the RAS [36]. In the present study, renal medullary and urinary renin activity was activated following AngII infusion, whereas plasma and renal cortical renin activity was suppressed, highlighting differences between systemic versus intrarenal renin systems as documented by previous studies [16, 18, 27]. AngII-induced increases in renal inner medullary and urinary renin activity were remarkably suppressed by IM PRO20. These results represent strong in vivo evidence for a role of PRR in the regulation of local renin activity during AngII-induced hypertension.

We assessed the direct role of PRR in the regulation of renin activity in cultured VSMC following AngII treatment. Exposure of VSMC to AngII induced a significant increase in medium renin activity, suggesting a positive feedback regulation of local RAS by AngII in the vasculature contrasting to the negative feedback regulation at the juxtaglomerular apparatus. This finding is in agreement with the appreciated role of the local RAS in the vascular remodeling in animal models of balloon injury [37], spontaneously hypertensive rats [38], and one-kidney, one-clip-induced and two-kidney, one-clip-induced hypertension [39, 40]. We found that the AngII-induced local renin response in VSMC was attenuated by PRO20, indicating involvement of PRR. Likewise, PRR plays an important role in amplifying the vascular renin response to AngII. We suspect the PRR-dependent activation of the local RAS may participate in the regulation of vascular function or remodeling during AngII-induced hypertension. This notion is in agreement with the significant role of PRR in determining the integrity of VSMC [41]. Besides VSMC, the CD is another important site for increased renal PRR expression in this hypertension model, as shown previously [16, 18], and likely plays a contributory role as well. Similarly, renin secretion from the CD cells is also stimulated by AngII [27] and this stimulation is likely mediated by PRR. The relative importance of vascular versus tubular PRR remains elusive and awaits genetic validation in the future studies. There is an intriguing possibility that PRR-dependent regulation of the local renin response may coordinate the functions of the vasculature and the CD. Such coordination can be mediated by releasing sPRR, which acts in an autocrine or paracrine fashion.

Irrespective of the underlying mechanism, the present study has characterized PRO20 as a novel therapeutic approach for hypertension and kidney injury. However, the well-recognized developmental role of PRR may imply a safety concern with this approach. In both low vertebrates and mammals, PRR plays an essential role in embryogenesis, likely via activation of the Wnt/β-catenin pathway [15, 36]. In particular, deletion of PRR in mice in a conventional or conditional manner leads to a lethal phenotype [36, 42]. However, to our surprise, there was no noticeable toxicity associated with PRO20 in the current experimental model. In agreement with this observation, inhibition of the Wnt/β-catenin pathway exhibits antifibrotic and protective effects in animal models of diverse fibrotic diseases in the kidney [43–45], skin [46], and lung [47] with a generally optimal safety profile. It is possible that the developmental pathway may be selectively activated in some disease processes and may represent an attractive therapeutic target.

## Conclusion

The present study employed a newly developed PRR-decoy peptide, PRO20, coupled with an intramedullary infusion technique to investigate the functional role of renal medullary PRR during AngII-induced hypertension. Not only did we demonstrate a remarkable BP-lowering effect of intramedullary PRR antagonism, but we also underscored a novel mechanism of this phenomenon involving PRR-dependent activation of the local renin response. We for the first time demonstrate the renin regulatory function of PRR in vivo and in vitro and report PRO20 as a novel therapeutic agent for hypertension and chronic kidney disease.

## Abbreviations

ACE: angiotensin-converting enzyme
AngII: angiotensin II
BP: blood pressure
CD: collecting duct
ELISA: enzyme-linked immunosorbent assay
IM PRO20: intramedullary PRO20 infusion
IV PRO20: intravenous PRO20 infusion
MAP: mean arterial pressure
PRR: (pro)renin receptor
qRT-PCR: quantitative reverse transcriptase polymerase chain reaction
RAS: renin-angiotensin system
sPRR: soluble form of PRR
VSMC: vascular smooth muscle cells
